# Comparative genomic analysis of *Mycobacterium avium *subspecies obtained from multiple host species

**DOI:** 10.1186/1471-2164-9-135

**Published:** 2008-03-20

**Authors:** Michael L Paustian, Xiaochun Zhu, Srinand Sreevatsan, Suelee Robbe-Austerman, Vivek Kapur, John P Bannantine

**Affiliations:** 1 National Animal Disease Center, USDA-ARS, Ames, USA; 2 Center for Animal Health and Food Safety, University of Minnesota, St. Paul, USA; 3 Department of Veterinary and Biomedical Sciences, Penn State University, University Park, USA

## Abstract

**Background:**

*Mycobacterium avium *(*M. avium*) subspecies vary widely in both pathogenicity and host specificity, but the genetic features contributing to this diversity remain unclear.

**Results:**

A comparative genomic approach was used to identify large sequence polymorphisms among *M. avium *subspecies obtained from a variety of host animals. DNA microarrays were used as a platform for comparing mycobacterial isolates with the sequenced bovine isolate *M. avium *subsp. *paratuberculosis *(MAP) K-10. Open reading frames (ORFs) were classified as present or divergent based on the relative fluorescent intensities of the experimental samples compared to MAP K-10 DNA. Multiple large polymorphic regions were found in the genomes of MAP isolates obtained from sheep. One of these clusters encodes glycopeptidolipid biosynthesis enzymes which have not previously been identified in MAP. *M. avium *subsp. *silvaticum *isolates were observed to have a hybridization profile very similar to yet distinguishable from *M. avium *subsp. *avium*. Isolates obtained from cattle (n = 5), birds (n = 4), goats (n = 3), bison (n = 3), and humans (n = 9) were indistinguishable from cattle isolate MAP K-10.

**Conclusion:**

Genome diversity in *M. avium *subspecies appears to be mediated by large sequence polymorphisms that are commonly associated with mobile genetic elements. Subspecies and host adapted isolates of *M. avium *were distinguishable by the presence or absence of specific polymorphisms.

## Background

*Mycobacterium avium *(*M. avium*) subspecies represent a closely related group of mycobacteria that are commonly found in the environment; some of which are frequently associated with infections of birds and ruminants. The *M. avium *subspecies are distinguished from each other by nucleic acid hybridization [1,2] along with growth characteristics [3]. *M. avium *subspecies *paratuberculosis *(MAP) is the causative agent of Johne's disease, a chronic and economically significant infection primarily of ruminant animals characterized by a prolonged subclinical phase leading eventually to a severe gastroenteritis which results in malnutrition and ultimately death. Much remains to be elucidated regarding the pathogenesis and population genetics of MAP. Specifically, it remains unclear what MAP virulence factors are important for both infection and persistence, and despite observed phenotypic and genetic differences between MAP isolates obtained from sheep and cattle, the biological basis for host specificity remains unclear.

Previous work in our laboratory has utilized DNA microarrays to compare the genome content of members of the *M. avium *complex (MAC) which includes MAP, *M. avium *subspecies *avium *(MAA), *M. avium *subspecies *silvaticum *(MAS), *M. avium *subspecies *hominissuis *(MAH) and *M. intracellulare *[4]. These findings revealed that non-MAP MAC isolates do not contain several large regions of genomic DNA that are present in MAP K-10. Extensive genomic conservation was observed for the MAP isolates examined in the study, most of which were obtained from cattle [4]. Recently, Marsh and coworkers have described the presence of several large sequence polymorphisms among sheep and cattle isolates of MAP [5,6], while Semret *et al *have reported on the presence of polymorphic regions that are shared between MAA and MAP sheep isolates [7]. The biological consequence of these large sequence polymorphisms has not yet been determined.

In the work presented here, we have utilized a DNA microarray constructed with oligonucleotides representing all of the predicted coding and intergenic regions from the MAP K-10 genome as well as the remaining novel coding sequences from the MAH 104 [8] genome to examine the genome content of *M. avium *subspecies obtained from a variety of host animals. We hypothesize that genes found to be polymorphic among *M. avium *subspecies isolated from different hosts will serve as ideal targets for future studies designed to elucidate the biological basis of host specificity and pathogenicity.

## Results

### Validation of microarray sensitivity and specificity

**Figure 1:**
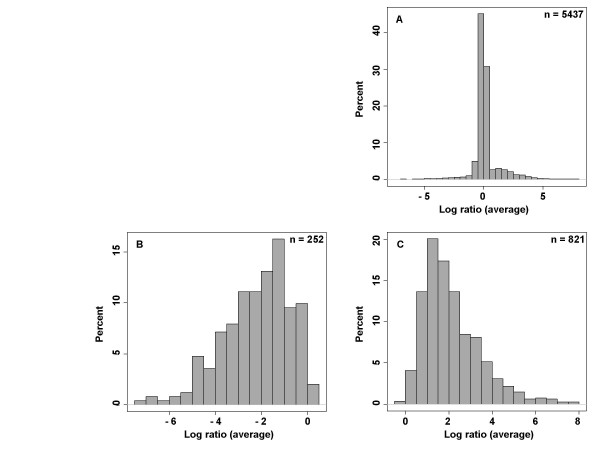
**Distribution of log transformed microarray fluorescence intensity ratios for competitive genomic hybridizations of MAH 104 and MAP K-10 (MAH 104/MAP K-10).** The results presented (n = number of results) are the average values of multiple hybridizations and replicate spots as described in Material and Methods. Log ratio distributions are displayed for (A) all probes present on the microarray; (B) MAP K-10 specific probes (as determined by BLAST E-value < 1 × 10^-5^); and (C) MAH 104 specific probes.

The reference isolate MAP K-10 was compared to the sequenced MAH isolate 104 in order to evaluate the performance of the oligonucleotide microarray as a platform for comparative genomic hybridizations. The hybridizations and data analyses were performed as described in the Materials and Methods, and the resulting intensity ratios for each oligonucleotide probe were matched to the BLASTn score of the probe when searched against the complete genomes of MAP K-10 and MAH 104. Probe sequences with an E-value of greater than 1 × 10^-5 ^were identified as not present in the reference genome. The distribution of log transformed hybridization intensity ratios (MAH 104/MAP K-10) is presented in Figure 1. A majority of the probes (75%) were observed to have ratios falling between -0.5 and 0.5, as would be expected for the genomes of these closely related mycobacteria. The distribution of MAP K-10 specific probes peaked below a log ratio of -1.0, with a majority of the probes having ratios lower than this. Mirroring this trend, the MAH 104 specific probes peaked above a log ratio of 1.0 with most of the probes observed to have ratios greater than this. Only five of the MAP K-10 specific probes had positive log ratios, while only three of the MAH 104 specific probes had negative log ratios. In both cases, the log ratios of these outlying probes fell within the range of -0.5 to 0.5. Based on these reference hybridizations, we predict that probes on the MAP K-10 microarray with log ratios greater than 1.2 can be identified as absent from the MAP K-10 genome, while probes with log ratios less than -1.2 are missing from the test isolates relative to MAP K-10. Utilizing these cutoffs, both the false positive and false negative rates are less than 1%.

### Sheep genomotype

**Figure 2:**
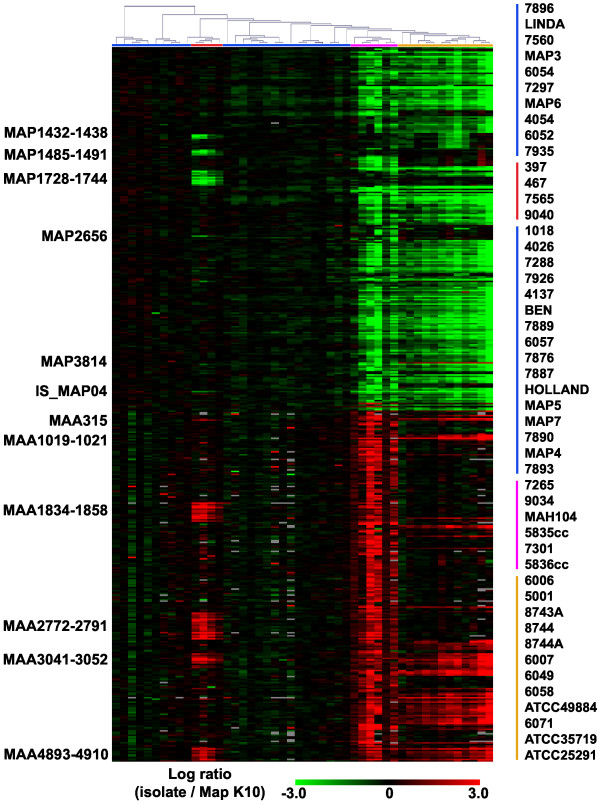
**Most variable ORFs amongst mycobacterial isolates examined.** 474 ORFs with 3-fold or greater differences in hybridization between at least two isolates and MAP K-10 are displayed in rows. Columns represent individual mycobacterial isolates that were grouped by hierarchical clustering (Pearson correlation, average linkage). Grey colored data points represent no data. Isolate designations are listed in the right margin next to a colored bar indicating the location of the isolates on the graph. Selected gene clusters are labeled by the first and last ORFs. A complete listing of the ORFs and log ratios has also been provided [see Additional file 1].

A similar genomic profile was observed for MAP sheep isolates 397, 467, 7565, and 9040. These sheep isolates were distinguished from the other MAP isolates examined in this study by the presence of four clusters of ORFs homologous to sequences found in the MAH 104 genome but absent in MAP K-10. Additionally, these isolates are missing three clusters of ORFs that are present in the other MAP isolates examined. The sheep isolate 9040 also appears to be a member of this genomotype, although the observed differences in hybridization intensity compared to MAP K-10 are not as pronounced as the other isolates (Figure 2).

Consistent with previous findings, we identified several regions that were present in MAP sheep isolates and MAH, but not in MAP cattle isolates. These included the MAH 104 ORFs MAH1834-1858 (Figure 2). This region was partially identified as the 197 bp sequence PIG-RDA 20 (AY266301) and was mapped to a 197 Kbp segment of the MAH 104 genome by Dohmann and coworkers [9] and was subsequently described by Semret and coworkers as LSP^A^4-II [7] (Table 1). This region contains a copy of the IS1311 insertion sequence (MAH1844) and within the MAH 104 genome is flanked by an additional copy of IS1311 (MAH1833). Another previously described LSP included ORFs MAH4893 through MAH4910 (Table 1). This region was partially identified as the 233 bp sequence PIG-RDA10 (AY266300), mapped to a 16 Kbp segment of the MAH 104 genome [9] and the full sequence was later identified as LSP^A^18 [7].

**Table 1:** *Mycobacterium avium *large sequence polymorphisms. Genomotypes from the present study that contain the indicated sequences are listed. Supporting evidence from previous studies is provided and includes the designations used to describe individual polymorphisms.

**ORF start**	**ORF end**	**Start**^b^	**End**^b^	**Present study**	**Dohmann 2003**	**Semret 2004**	**Talaat 2006**	**Semret 2006**
MAH0238		254394	294226	HOM		LSPA 3	MAV-1	
MAH0439		461414	492800	HOM		LSPA 7	MAV-2	
MAH0636		666033	675725	HOM		LSPA12	MAV-3	
MAH0722		747095	794450	HOM		LSPA 5	MAV-4	
MAH1019	MAH1021	1049356^a^		HOM, AV-SI				
MAH1354		1421722	1439626	HOM		LSPA 14	MAV-5	
MAH1374		1444205	1463365	HOM		LSPA 13	MAV-6	
MAH1663		1795281	1991691	HOM		LSPA 4	MAV-7	
MAH1834	MAH1858	1992430		HOM, MAP Sheep	PIG-RDA 20			LSPA 4-II
MAH1940		2097907	2100883	HOM			MAV-8	
MAH2053		2220320	2241163	HOM		LSPA 10	MAV-9	
MAH2085		2259120	2271610	HOM			MAV-10	
MAH2254		2462693	2466285	HOM			MAV-11	
MAH2331		2549555	2730999	HOM		LSPA 1	MAV-12	
MAH2591		2815625	2821149	HOM			MAV-13	
MAH2766		3008716	3036980	HOM			MAV-14	
MAH2772	MAH2791	3014473^a^		HOM, MAP Sheep	PIG-RDA 30			
MAH2829	MAH2830	3077414^a^		AV-SI				
MAH2916		3214820	3219550	HOM, AV-SI			MAV-15	
MAH3023	MAH3026	3340393	3384549	HOM, AV-SI			MAV-16	
MAH3034		3392586	3413804	HOM		LSPA 9	MAV-17	
MAH3041	MAH3052	3399659^a^		HOM, MAP Sheep, AV-SI				
MAH3156		3523417	3527334	HOM			MAV-18	
MAH3296		3670518	3675686	HOM			MAV-19	
MAH3542		3917752	3939034	HOM		LSPA 2	MAV-20	
MAH3858	MAH3866	4254594	4261488	HOM, AV-SI			MAV-21	
MAP4086	MAP4091	4674473	4682256	HOM, MAP, AV-SI		LSPA11		
MAH4657	MAH4665	5122371	5132301	HOM, AV-SI		LSPA 8	MAV-22	
MAH4704	MAH4709	5174641	5270187	HOM, AV-SI		LSPA 6	MAV-23	
MAH4893	MAH4910	5378903	5395102	HOM, MAP Sheep, AV-SI	PIG-RDA 10		MAV-24	LSPA 18

a Approximate.

b Nucleotide position within MAH 104 genome

Several new or only partially described LSPs common to MAP sheep and MAH isolates were identified in the present study. One of the genomic regions included ORFs MAH2772 – 2791 (Figure 2 and Table 1). This gene cluster was partially identified as the 548 bp sequence PIG-RDA 30 (AY266302) and was mapped to a 27 Kbp region on the MAH 104 genome [9]. This cluster of genes encodes several proteins that may contribute to pathogenesis. MAH2777 is predicted to encode a cytochrome P450 enzyme. Members of this enzyme family have been implicated in basic cellular processes as well as virulence (reviewed in [10]). MAH2782 is homologous to an arylsulfatase characterized in *Pseudomonas aeruginosa *and like other sulfatases may modulate mycobacterial interactions with host cells (reviewed in [11]). This gene cluster is also characterized by the presence of several genes encoding putative proteins involved in lipid and energy metabolism. Seven of the identified genes encode proteins with no predicted function.

**Figure 3:**
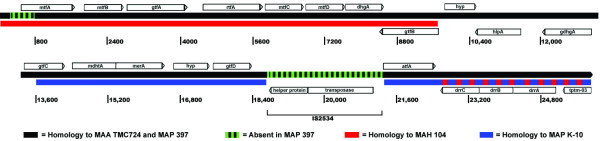
**Glycopeptidolipid biosynthesis gene cluster in MAP sheep isolate 397.** Solid black lines represent homology (approximately 99% identity) with the MAA TMC724 (ATCC 25291) glycopeptidolipid biosynthesis cluster (accession number AF125999), while dashed green lines represent sequences absent in isolate 397. Solid or dashed red and blue lines represent regions of homology with MAH 104 and MAP K-10, respectively. Vertical lines are spaced at 1600 base intervals for reference and represent the number of nucleotides from the start of the Genbank entry.

Another novel LSP found in MAP sheep and MAH isolates but absent from MAP cattle isolates was comprised of ORFs MAH3041 – 3052 (3,399,659 – 3,411,697) which are predicted to encode proteins involved in the biosynthesis of glycopeptidolipids [12,13] (Table 1). Sequencing of this region in the sheep isolate MAP 397 revealed the presence of additional ORFs (*hyp*, *hlpA*, *gdhgA *and *gtfC*) with homology to glycopeptidolipid biosynthesis genes immediately downstream of MAH3052 (*gtfB *in Figure 3). These additional ORFs were not homologous to any MAH 104 sequences (and thus were not detected by the microarray analysis) but rather were homologous to MAA TMC724 (ATCC 25291), a serovar 2 isolate of MAA. A direct comparison of the glycopeptidolipid biosynthesis gene clusters from MAP 397 and MAA TMC724 (accession number AF125999) revealed that they are approximately 99% identical with the exception of a deletion at the 5' end of mtfA as well as the absence of the insertion sequence IS2534 from the MAP 397 locus (Figure 3). Despite the presence of these genes, no glycopeptidolipids have been observed to be produced by sheep isolates of MAP under standard *in vitro *culture conditions (T. Eckstein, personal communication); therefore, the practical contribution of glycopeptidolipids to the pathogenesis of MAP sheep isolates remains unclear at this point.

A putative transcriptional regular labeled as MAH315 in MAH 104 was identified by microarray hybridization as present in MAP sheep isolates. This ORF was subsequently confirmed as present in the MAP 397 genome by sequencing. The protein encoded by MAH315 has homology to the GntR-family of transcriptional regulators which are widely distributed across bacterial species and regulate a variety of cellular processes [14,15].

A second subset of LSPs were characterized as being present in all MAP isolates with the exception of the sheep genomotype. Several of these MAP sheep isolate deletions have already been described. The deletion encompassing MAP1485c – 1491 was previously identified by Marsh and coworkers as S strain deletion #1 and by Semret and coworkers as LSP^A^20 [5,7] (Table 2). An additional deletion in the sheep genomotype included the cluster of ORFs between MAP1728c and MAP1744 (Table 2). This deletion was partially identified by Marsh and coworkers as RDA3 [6], and later fully described as S deletion #2 [5]. MAP2325 was identified as being absent from Australian sheep isolates of MAP [5]. This ORF was not identified as missing from the MAP sheep isolates examined in this study, and subsequent sequencing of this region in MAP 397 confirmed the presence of an ORF with 100% identity to MAP2325. Although further examination of this observation is warranted, this discrepancy may represent a geographic difference between MAP isolates recovered from sheep in Australia and the United States.

**Table 2:** *Mycobacterium avium *large sequence polymorphisms. Genomotypes from the present study that lack the indicated sequences are listed. Supporting evidence from previous studies is provided and includes the designations used to describe individual polymorphisms. ID numbers correspond to Figure 4.

**Number**	**ORF start**	**ORF end**	**Present study**	**Tizard 1998**	**Dohmann 2003**	**Paustian 2005**	**Semret 2005**	**Marsh 2005**	**Marsh 2006**	**Talaat 2006**	**Semret 2006**
	MAP0072c	MAP0076	HOM (5835cc, 5836cc)								
1	MAP0092	MAP0108	HOM, AV-SI			MAP_RD1	LSPP1			MAP-1	
2	MAP0282c	MAP0284c	HOM, AV-SI				LSPP2			MAP-2	
3	MAP0387	MAP0389	HOM, AV-SI				LSPP3			MAP-3	
4	MAP0746	MAP0766c	AV-SI (6007, 6049)								
5	MAP0850c	MAP0866	HOM, AV-SI		RDA I130	MAP_RD2	LSPP4			MAP-4	
6	MAP0956	MAP0967	HOM				LSPP5			MAP-5	
7	MAP1230	MAP1237c	HOM	GS			LSPP6			MAP-6	
8	MAP1344	MAP1349c	HOM, AV-SI				LSPP7			MAP-7	
9	MAP1376c	MAP1381	AV-SI								
10	MAP1424c	MAP1465	AV-SI (6007, 6049, 6058, ATCC49884)								
11	MAP1432	MAP1438c	MAP Sheep								
12	MAP1485c	MAP1491	MAP Sheep						S 1		LSPA20
13	MAP1631c	MAP1638c	HOM				LSPP8			MAP-8	
14	MAP1718c	MAP1727	HOM		RDA III10		LSPP9			MAP-9	
15	MAP1721c	MAP1723	HOM, AV-SI								
16	MAP1728c	MAP1744	MAP Sheep					RDA3	S 2		
17	MAP1729c	MAP1732c	AV-SI				LSPP9			MAP-10	
18	MAP2026	MAP2029c	HOM				LSPP10			MAP-11	
19	MAP2148	MAP2158	HOM, AV-SI			MAP_RD3	LSPP11			MAP-12	
20	MAP2178	MAP2196	HOM, AV-SI			MAP_RD4	LSPP12			MAP-13	
21	MAP2325	MAP2325							S 3		
22	MAP2372	MAP2375c	AV-SI (5001, 6006, 6058, ATCC49884)								
	MAP2523c	MAP2532	HOM (5835cc, 5836cc)								
23	MAP2751	MAP2769c	HOM, AV-SI			MAP_RD5	LSPP13			MAP-14	
24	MAP2963c	MAP2965c	HOM, AV-SI			Unnamed				MAP-15	
25	MAP3067c	MAP3079c	AV-SI (5001, 6006, 6058, ATCC49884)								
26	MAP3721	MAP3776c	HOM, AV-SI			MAP_RD6	LSPP14/15			MAP-16	
27	MAP3814	MAP3818	HOM		RDA II60	MAP_RD7	LSPP16				
28	MAP4266	MAP4270	HOM, AV-SI				LSPP17			MAP-17	MAA, MAS
29	MAP4326c	MAP4328c	HOM							MAP-18	

A novel LSP comprised of the ORFs MAP1432 – 1438c was identified in the present study as absent from sheep MAP isolates (Table 2). This gene cluster is predicted to encode four energy metabolism enzymes as well as a lipase (MAP1438c). MAP1432 encodes a hypothetical protein with homology to the REP13E12 family of repetitive elements that were originally described in *Mycobacterium tuberculosis *and have been shown to be targets of phage integration [16]. MAP2656 was initially identified as absent via microarray analysis but sequencing of MAP 397 identified a homologue with 100% identity. This represents the only observed discrepancy between the microarray and sequencing results.

### Avium-silvaticum genomotype

The genome profile of MAP sheep isolate 5001 was unlike the other isolates obtained from sheep that were examined in this study. This isolate grouped together with four independent laboratory stocks of what has historically been referred to as *M. avium *strain 18 [17-22] as well as five isolates of MAS (Figure 2). Additionally, two isolates of MAA (ATCC25291 and ATCC35719) were observed to have genomic profiles similar to this group. The hybridization pattern of these isolates is collectively referred to here as the avium-silvaticum genomotype.

Similar to the MAP sheep isolates, the avium-silvaticum genomotype isolates contained LSPs that are absent from MAP cattle isolates but present in MAH 104. They possessed the MAH3041-3052 and MAH4893-4910 gene clusters also found in MAP sheep isolates; however, they did not possess the MAH1834-1858 or MAH2772-2791 clusters of MAH 104 ORFs (Figure 2 and Table 1). These isolates contained additional LSPs homologous to the MAH 104 genome. A cluster of MAH 104 ORFs including MAH4657-4665 (5,122,587–5,131,447) was present in the avium-silvaticum genomotype isolates. The ORFs in this LSP are predicted to encode proteins that function as transcriptional regulators and dehydrogenases, while several have no known function. A second LSP with homology to the MAH 104 genome included ORFs MAH4704-4709 (5,173,587–5,179,816) (Table 1). With the exception of two ORFs predicted to encode components of a restriction modification system, the remaining coding sequences present in this LSP have no predicted function.

Several LSPs comprised of MAP K-10 ORFs were absent from the avium-silvaticum genomotype isolates (Figure 2). In many cases, the avium-silvaticum genomotype isolates were distinguished from each other by these LSPs. Isolates 6007 and 6049 lack a cluster of ORFs between MAP0746 and MAP0766c (Figure 2 and Table 2). This LSP was present in all of the other isolates examined and verified in selected isolates by PCR (Table 3). Included within this LSP are nine ORFs homologous to mammalian cell entry gene clusters [23,24]. The other ORFs in this cluster encoded degradation enzymes or had no predicted function. The avium-silvaticum genomotype isolates were also missing ORFs MAP1376-1381, which were also absent in the hominissuis genomotype isolates 5835cc and 5836cc described below (Table 2). The absence of this region was verified by PCR with primers designed for representative ORFs (Table 3). The MAS isolates 6007, 6049, 6058, and ATCC49884 were distinguished from all the other isolates examined by the absence of ORFs MAP1424c-1464 (Table 2). This LSP includes a gene cluster that was identified as absent from the sheep genomotype (MAP1432-1438c). A large number of the ORFs included in this region (n = 16) are predicted to be involved in lipid metabolism. Notably, just as the smaller sheep isolate LSP was flanked by a REP13E12 family repetitive element (MAP1432), the larger MAS LSP is also flanked by a REP13E12 element (MAP1465) in MAP K-10. The isolates 5001, 6006, 6058, and ATCC49884 lack the ORF clusters MAP2372-2375c and MAP3063-3079c (Table 2). These LSPs were present in all of the other isolates examined and contain ORFs primarily encoding proteins with no predicted functions, although MAP3069-3071 encode proteins that are predicted to be involved in terpenoid biosynthesis. MAH2829-2830 were identified as present only in isolates ATCC35719, ATCC25291, and 6071 as well as the strain 18 isolates.

**Table 3:** PCR amplification results for selected ORFs and isolates

	**Mycobacterial Isolates**					
**ORF**	**K-10**	**397**	**MAH104**	**5001**	**6007**	**6058**
MAP0025	+^a^	+	+	-^b^	-	-
IG130	+	+	+	+	+	-
MAP0745c	+	+	-	+	+	+
MAP0750c	+	+	+	+	-	+
MAP1064	+	+	+	-	-	-
MAP1375c	+	+	+	+	+	+
MAP1379	+	+	+	-	-	-
MAP1423	+	+	+	-	-	-
MAP1431	+	+	+	-	-	-
MAP1439c	+	+	+	-	-	-
MAP1720	+	+	-	+	+	+
MAP1821c	+	+	+	-	-	-
IG1617	+	+	+	+	+	-
MAP2374c	+	+	+	-	+	-
MAP3066c	+	+	+	-	+	-
MAP3072	+	+	+	-	+	+
MAP3437c	+	+	+	-	-	+
MAP3540c	+	+	+	-	-	-
MAP3998c	+	+	+	+	+	-
MAP4079	+	+	+	+	+	+
MAP4333	+	+	+	+	-	-
MAH0315	-	+	+	+	+	+
MAH1018	+	+	+	+	+	+
MAH1020	-	-	+	+	+	+
MAH1368	-	+	+	-	-	-
MAH1850	-	+	+	-	-	-
MAH2252	-	-	+	-	-	-
MAH2336	-	-	+	+	+	+
MAH3231	-	-	+	+	+	+
MAH3297	-	-	+	+	+	+
MAH3358	-	-	+	+	+	+
MAH4015	-	-	+	+	+	+
MAH4520	-	-	+	+	+	+
MAH4778	-	-	+	-	-	-

a PCR product detected.

b PCR product with reduced yield or not detected

All of the avium-silvaticum genomotype isolates were also observed to have greater amounts of probe hybridized to the oligonucleotide target representing MAP3814c relative to MAP K-10 (Figure 2). This ORF is 31% identical to transposase sequences and may represent part of an insertion sequence that is present in multiple copies within MAS and MAA genomes. The MAP K-10 ORFs immediately downstream of MAP3814c (MAP3815-3818) are missing in both the avium-silvaticum and hominissuis genomotype isolates based on the microarray hybridization results. Notably, ORFs MAP3815-3817 lack homology to any other sequences deposited in Genbank. This appears to provide an additional example of mycobacterial genome diversity mediated by a mobile genetic element.

The avium-silvaticum genomotype isolates could be further separated into two subgroups based on their genomic hybridization profiles. One group included the isolate 5001 and the isolates originally identified as MAS, while the other consisted of the MAA and strain 18 isolates, which the exception of isolate 6071. While both groups of isolates displayed very similar hybridization profiles, they could be distinguished by the polymorphic regions MAP1424c-1464 and MAH2829-2830. The consistent genomic profile amongst the strain 18 isolates obtained from independent laboratories suggests that these isolates have not experienced significant genomic polymorphisms despite several decades of laboratory use. Additionally, these results indicate that the microarray platform used in this study provides reproducible results when comparing isolates.

### Hominissuis genomotype

Three of the MAP isolates examined (7265, 7301, and 9034) had hybridization profiles similar to MAH 104 and are collectively referred to as the hominissuis genomotype. Also included with these isolates were two mycobacterial isolates (5835cc and 5836cc) from a duck, which were distinguished from each other upon culture by pigmentation. This group is characterized by the absence of several genes and LSPs that are present in MAP, as well as the presence of additional LSPs not found in MAP. Many of these LSPs are shared with the MAS and MAA isolates examined in this study. The sequenced MAP K-10 and MAH 104 genomes have been previously compared with both bioinformatic and experimental approaches, including DNA microarrays [4,25-28]. The LSP that includes ORFs MAP1230-1237c appears to be a distinguishing factor of the hominissuis genotype as these ORFs were absent from this genomotype but present in all of the other isolates examined in this study (Table 1). This LSP corresponds to the region previously identified by Tizard and coworkers as a low-GC genetic island present only in MAP and MAS [29]. Isolates 5835cc and 5836cc were distinguished from the other hominissuis genomotype isolates by several polymorphic regions. MAP0072c-0076 is absent from both isolates and includes several genes that are predicted to encode membrane proteins, while the deletion of MAP2523c-2532 includes several enzymes involved in energy metabolism (Figure 2). The hybridization profiles of the remaining hominissuis genomotype isolates closely mirrored MAH 104.

### Cattle genomotype

The remaining MAP isolates examined in this study displayed hybridization profiles similar to the sequenced cattle isolate MAP K-10 and are collectively referred to as the cattle genomotype. The distinguishing feature of this genomotype is the large variety of host species represented. This group includes isolates obtained from cattle (n = 5), birds (n = 4), goats (n = 3), bison (n = 3), humans (n = 9), a cat, and an armadillo (Figure 2). Relative to the MAP K-10 genome, no LSPs were observed in any of the cattle genomotype isolates.

## Discussion

The use of specific genetic markers has allowed MAP isolates to be separated into two general populations: a relatively homogenous group comprised of primarily bovine isolates and a more heterogeneous group that includes isolates from small ruminants and other mammals. The goal of the present study was to identify variations in total genome content between *M. avium *subspecies isolated from several different host animals in order to determine which genes may contribute to host specificity and pathogenesis. The isolates obtained from goat (n = 3), bison (n = 3), bird (n = 4), armadillo (n = 1), cat (n = 1), and human (n = 10) hosts did not contain any large polymorphic regions when compared with the MAP K-10 cattle isolate. Five of the seven cattle isolates examined similarly did not contain any large polymorphisms. The remaining isolates (n = 7) grouped into three distinct genomotypes. Four of the six sheep isolates examined (397, 467, 7565 and 9040) shared large polymorphisms that included the absence of ORFs present in the MAP K-10 genome as well as the presence of ORFs that are also found in the MAH 104 genome. The ORFs included in the missing regions (n = 32) are predicted to encode proteins involved in a variety of functions including lipid and energy metabolism, virulence, and transcriptional regulation. The regions containing homologues to ORFs in the MAH 104 genome (n = 73) encoded proteins involved in glycopeptidolipid biosynthesis, transcriptional regulation, virulence, and metabolism. These polymorphic regions also contained a number of proteins with unknown function. The remaining sheep (5001 and 9034) and cattle (7265 and 7301) MAP isolates had genomic profiles similar to MAH or MAS.

The identification of a glycopeptidolipid biosynthesis operon in sheep isolates of MAP raises several interesting possibilities. Considering the absence of glycopeptidolipids in cattle isolates of MAP, it may contribute to the phenotypic differences observed between the two isolate types, although the production of glycopeptidolipids by sheep MAP isolates has not yet been experimentally verified. Glycopeptidolipids from MAA have been identified as Toll-like receptor 2 agonists that are capable of activating macrophages [30]. Sequencing of this region indicates that the sheep isolate glycopeptidolipid biosynthesis cluster is most similar to a serovar 2 isolate of MAA (ATCC 25291) rather than the sequenced serovar 1 isolate MAH 104. ATCC 25291 was examined as part of this study, however, since no sequence data from this isolate was present on the microarray, the presence or absence of these genes was not detected by hybridization. These results suggest that additional insights into the pathogenicity and evolutionary history of MAP sheep isolates may be gained by a closer examination of MAA serovar 2 genomes.

This study provides some of the first molecular evidence that distinguishes MAS from other *M. avium *subspecies. The MAS and MAA isolates examined in this study were observed to have very similar genomic hybridization profiles, but could be distinguished by several large sequence polymorphisms. Specifically, the *silvaticum *and *avium *subspecies could generally be separated from each other by the presence or absence of the polymorphic regions MAP1424c-1465 and MAH2829-2830. Isolate 5001 was grouped together with the classical MAS wood pigeon isolates. This isolate was originally isolated from a sheep that appeared to be suffering from Johne's Disease. It is noteworthy that in a previous study by Burrells and coworkers MAS was also cultured from sheep that were diagnosed with Johne's Disease [31]. The silvaticum genomotype isolates share some distinguishing genome features with the MAP sheep isolates, including all or part of three LSPs. It is unclear whether the recovery of MAS isolates from diseased animals is due to the increased susceptibility of sheep to MAS infections or if the inherent difficulties of culturing sheep MAP isolates *in vitro *results in the isolation of secondary opportunistic pathogens. Regardless, the association of MAS with infections resembling Johne's Disease warrants additional study.

**Figure 4:**
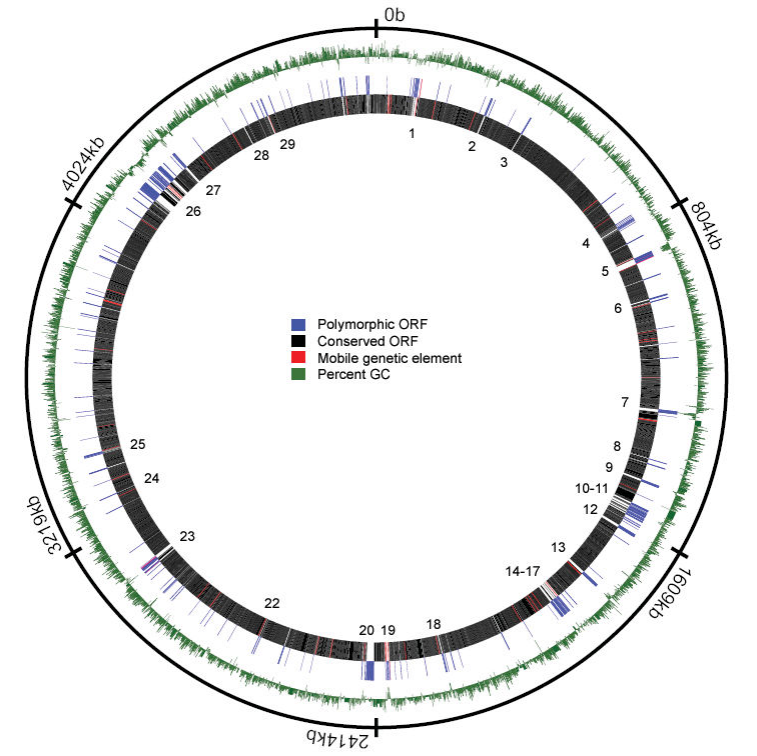
**Location of polymorphic regions mapped to the MAP genome.** The outermost ring is the nucleotide position from the origin of replication, while the green ring is the percentage of GC nucleotides. The innermost rings represent the ORFs that were identified as variably present (log ratio < -1.2) in at least one of the mycobacterial genomes (blue) as well as ORFs conserved in all of the isolates examined (black). ORFs that are annotated as mobile genetic elements are colored red. The numbers adjacent to polymorphic sequences correspond to the LSP numbers listed in Table 2. The circular genome MAP was generated with the software program Microbial Genome Viewer [50].

The LSPs observed in the mycobacterial isolates were often closely associated with mobile genetic elements and a reduction in the proportion of GC-base pairs relative to the MAP genome average of 69.3% (Figure 4). A majority of the LSPs contain or are flanked by insertion sequences, phage remnants, or REP-family elements, which themselves have been predicted to be sites of phage integration. Considering the paucity of recombination observed amongst mycobacteria, it is not surprising that much of the sequence diversity amongst this closely related group of mycobacteria appears to be localized to loci that are highly mobile and hotspots for recombination.

It is notable that two of the LSPs include *mce *gene clusters. The *mce *genes were originally described in *M. tuberculosis *as facilitating mycobacterial cell entry [32] and subsequent genome sequencing identified four *mce *genes clusters in the *M. tuberculosis *genome (*mce1*, *mce2*, *mce3*, and *mce4*) [33]. Deletion of the *mce *gene clusters in *M. tuberculosis *resulted in the attenuation of virulence in mouse models [34,35]. Recent work has suggested that the *mce *gene clusters may function as ATP-binding cassette transport systems [36] with cholesterol subsequently identified as one potential substrate [37]. A phylogenetic analysis of the *mce*-like genes from a variety of microbes further supports the predicted function of these gene clusters as ABC type transporters [38]. Analysis of the MAP genome sequence has identified eight mce gene clusters [39]. The two *mce *gene clusters identified as polymorphic in the isolates examined in this study (MAP0757-0765 and MAP2189-2194) were annotated by Casali and Riley as *mce5 *and are predicted to have arisen from a recent gene duplication event [38]. The MAP2189-2194 *mce5 *cluster was absent from most hominissuis and avium-silvaticum genomotype isolates, while the MAP0757-0765 cluster was only absent from MAS isolates 6007 and 6049.

While the LSPs that distinguish cattle and sheep MAP isolates represent a potential source of species specificity, the lack of LSPs amongst the cattle genomotype isolates suggests that other mechanisms may also have an influence. Alternatively, the variety of species from which the cattle genomotype isolates were recovered may represent transient or "pass through" infections by the more environmentally prevalent cattle type isolates. From a human health perspective, all of the human MAP isolates examined in this study appear to be highly similar to the prototypical bovine isolates. While the source and route of MAP infections in humans remains controversial, it appears that both cattle and humans are susceptible to infection by MAP isolates with similar genotypes. The diversity of SSR genotypes that displayed similar hybridization patterns suggests that additional insights into host specificity may be gained by future studies designed to examine small nucleotide changes.

The microarrays used in this study are not sensitive enough to detect small genetic changes such as single nucleotide polymorphisms (SNPs). Alternate approaches such as directed sequencing are required to elucidate these types of genetic differences which may also result in significant biological effects. Additionally, only sequences present on the microarray can be detected via hybridization, thus novel sequences that may be present in other mycobacterial genomes will not be observed. This limitation was partially addressed by sequencing portions of the MAP sheep isolate 397 genome. Genome sequencing and subtractive genomic approaches are warranted in future studies to identify additional sequences not represented on the MAP microarray used in this study.

## Conclusion

This study has identified several polymorphic regions within the genomes of *M. avium *subspecies obtained from a variety of host animals. Many of the subspecies are distinguished from each other by both host specificity as well as large sequence polymorphisms, which suggests that genes encoded within these regions influence the host range of the mycobacteria. The lack of genetic diversity and widespread distribution of cattle genomotype isolates of MAP suggest that this subspecies is either capable of infecting a variety of host species or alternatively is widespread in the environment and therefore comes into transient contact with a large number of hosts. Future experiments will be designed to elucidate the functions of the genes contained within the large sequence polymorphisms. Insights into the variable pathogenicity and host specificity of the closely related *M. avium *subspecies are likely contained within these regions.

## Methods

### SSR Genotyping

PCR amplification of two of the most discriminatory short sequence repeat (SSR) loci – mononucleotide G and tri-nucleotide GGT repeats [40] was carried out as described (2, 30) using primer sets (i) 5'-TCA GAC TGT GCG GTA TGG AA-3' and 5'-GTG TTC GGC AAA GTC GTT GT-3', and (ii) 5'-AGA TGT CGA CCA TCC TGA CC-3' and 5'-AAG TAG GCG TAA CCC CGT TC-3', respectively. The PCR products were purified with a QIAquick PCR purification kit (Qiagen Inc., Valencia, CA) and sequenced by using standard dye terminator chemistry, and the sequences were analyzed on an automated DNA sequencer (3700 DNA Analyzer, Applied Biosystems, Foster City, CA). The alleles were assigned a number congruent to the number of G and GGT residues.

### Polymerase chain reaction

All primers were designed with Primer3 software [41]. PCR reactions used genomic DNA as a starting template and used standard conditions reported in detail elsewhere [4].

### Microarray Design

The software program ArrayOligoSelector [42] was used to identify 70 mer oligonucleotides specific for every predicted open reading frame (ORF) in the MAP K-10 genome [39]. One 70 mer was designed for each MAP K-10 ORF with a total length of less than 4000 bp, while longer ORFs were split in half and one 70 mer was designed for each half. One 70 mer was also designed for every MAP intergenic region greater than 500 bp. Additionally, an automated annotation of the unannotated MAH 104 genome sequence (downloaded on 03/25/05) was performed using the methods described by McHardy and coworkers [43] and one 70 mer was designed for every predicted ORF that was less then 30% identical to MAP K-10 sequences as determined by BLAST analysis [44]. The annotated MAH 104 ORFs were numbered sequentially starting at the origin of replication, although for reference the nucleotide start and stop positions for each ORF or cluster are also reported. The arbitrarily assigned ORF designations were subsequently matched to the ORF designations used when the completed MAH 104 genome sequence was deposited in Genbank (accession number NC_008595) [see Additional file 1]. A detailed description of this microarray platform has been deposited in the National Center for Biotechnology Information (NCBI) Gene Expression Omnibus under the accession number GPL3433.

### Bacterial growth and DNA extraction

Mycobacteria were cultured and DNA extracted as previously described [45] or as follows. Bacteria were grown in Middlebrook 7H9 broth (pH 6.0) supplemented with oleic acid-albumin-dextrose-catalase (Becton Dickinson Microbiology, Sparks, MD), and 0.05% Tween 80. Cultures of MAP were further supplemented with ferric mycobactin J (2 mg/liter; Allied Monitor Inc., Fayette, MO). Genomic DNA was extracted from MAP isolates (Table 4) with Genomic-tip 100/G anion-exchange columns (QIAGEN, Valencia, CA) as previously described [46] with the following modification: D-cycloserine was not added as part of the extraction procedure. For the purposes of this study, *Mycobacterium avium *subsp. *hominissuis *is used to designate *Mycobacterium avium *isolates cultured from non-avian sources, while *Mycobacterium avium *subsp. *avium *is used for isolates originating from birds [8].

**Table 4:** Mycobacterial isolates used in this study.

			**SSR Type**						
**Isolate**	**Organism**^a^	**Host**	**G-rep**	**GGT-rep**	**IS1311 type**	**hsp65 PstI digest**	**Mycobactin J dependency**	**Source**	**Genomotype**^b^
7926	MAP	Armadillo	7	4	cattle	undigested	YES	UMN^c^	Cattle
7288	MAP	Bison	7	5	bison	undigested	YES	UMN	Cattle
7297	MAP	Bison	7	4	bison	undigested	YES	UMN	Cattle
7560	MAP	Bison	7	4	bison	undigested	YES	UMN	Cattle
7876	MAP	Cat	14	5	cattle	undigested	YES	UMN	Cattle
1018	MAP	Cattle	7	4	cattle	undigested	YES	UMN	Cattle
6052	MAP	Cattle					YES	UMN	Cattle
6054	MAP	Cattle					YES	UMN	Cattle
6057	MAP	Cattle					YES	UMN	Cattle
7265	MAP	Cattle	10	5	cattle	undigested	YES	ATCC 19815	Hominissuis
7301	MAP	Cattle	11	5	cattle	undigested	YES	UMN	Hominissuis
7935	MAP	Cattle	14	5	cattle	undigested	YES	UMN	Cattle
4026	MAP	Goat	10	5	cattle	undigested	YES	UMN	Cattle
4054	MAP	Goat	7	5	cattle	undigested	YES	UMN	Cattle
4137	MAP	Goat	12	5	cattle	undigested	YES	UMN	Cattle
7896	MAP	Human			cattle	undigested	YES	ATCC 43545	Cattle
BEN	MAP	Human	7	4	cattle	undigested	YES	ATCC 43544	Cattle
HOLLAND	MAP	Human	7	4	cattle	undigested	YES	ATCC 49164	Cattle
LINDA	MAP	Human	7	5	cattle	undigested	YES	ATCC 43015	Cattle
MAH104	MAH	Human			avium		NO	NADC^d^	Hominissuis
MAP3	MAP	Human	7	5	cattle	undigested		UMN	Cattle
MAP4	MAP	Human	7	4	cattle	undigested	YES	UMN	Cattle
MAP5	MAP	Human	7	5	cattle	undigested		UMN	Cattle
MAP6	MAP	Human	7	5	cattle	undigested	YES	UMN	Cattle
MAP7	MAP	Human	7	5	cattle	undigested		UMN	Cattle
397	MAP	Sheep			sheep		YES	NADC	Sheep
467	MAP	Sheep			sheep		YES	NADC	Sheep
5001	MAP	Sheep	2GC4G	3	sheep	digested	YES	UMN	Avium-silvaticum
7565	MAP	Sheep	15	3	sheep	undigested	YES	UMN	Sheep
9034	MAP	Sheep			sheep	undigested		UMN	Hominissuis
9040	MAP	Sheep			sheep	undigested		UMN	Sheep
7887	MAP	Starling	15	5	cattle	undigested	YES	UMN	Cattle
7889	MAP	Starling	7	5	cattle	undigested	YES	UMN	Cattle
7890	MAP	Starling	13	5	cattle	undigested	YES	UMN	Cattle
7893	MAP	Starling	12	5	cattle	undigested	YES	UMN	Cattle
6071	MAA	Strain 18					NO	NADC	Avium-silvaticum
8744	MAA	Strain 18	2GC4G	3				UMN	Avium-silvaticum
8743A	MAA	Strain 18	2GC4G	3				UMN	Avium-silvaticum
8744A	MAA	Strain 18	2GC4G	3				UMN	Avium-silvaticum
6006	MAS	Pigeon			avium		NO	NADC	Avium-silvaticum
6007	MAS	Pigeon					NO	NADC	Avium-silvaticum
6049	MAS	Pigeon					NO	NADC	Avium-silvaticum
6058	MAS	Pigeon			avium		NO	NADC	Avium-silvaticum
ATCC49884	MAS	Pigeon					NO	ATCC 49884	Avium-silvaticum
ATCC25291	MAA	Chicken					NO	ATCC 25291	Avium-silvaticum
ATCC35719	MAA	Chicken					NO	ATCC 35719	Avium-silvaticum
5835cc	MAA	Duck						NADC	Hominissuis
5836cc	MAA	Duck						NADC	Hominissuis

a Classification upon initial isolation.

b Genomotype is based upon the microarray hybridization profile.

c University of Minnesota – laboratory of Dr. Srinand Sreevatsan.

d National Animal Disease Center – laboratory of Dr. Michael Paustian

### Microarray hybridization and data analysis

Microarray hybridization and preliminary data filtering was performed as described in the National Center for Biotechnology Information (NCBI) Gene Expression Omnibus (GEO) database entries (see below). The software program Cluster 3.0 [47] was used to filter out ORFs that were lacking values for greater than 80% of the MAP isolates examined and median center the results for each isolate. Hierarchical clustering of the results was performed and visualized with Cluster 3.0 and Java TreeView [48] or MultiExperiment Viewer [49]. Putative large sequence polymorphisms (LSPs) were identified by a visual examination of the hybridization results and selected LSPs were subsequently confirmed by genome sequencing and/or PCR. All microarray data has been deposited in the NCBI Gene Expression Omnibus database as series number GSE7622 which includes sample accession numbers GSM172202 – GSM172344.

### Genome sequencing

Purified genomic DNA from MAP isolate 397 was prepared as described above for microarray hybridizations. Initial shotgun genome sequencing was performed by 454 Life Sciences (Branford, CT). Additional sequences were generated by directly sequencing PCR products and genomic DNA using an ABI3100 capillary sequencer (Applied Biosystems). Sequence data was generated as part of a genome sequencing project and will be published upon completion (M. Paustian, unpublished data).

## Authors' contributions

MP performed microarray hybridizations and genomic sequence data analysis. XZ cultured bacteria, isolated genomic DNA, and performed SSR typing and data analysis. SS provided bacterial isolates and analyzed phylogenetic data. SR performed bacterial isolations and data analysis. MP and SS wrote the manuscript. MP, SS, VK, and JB participated in the design of the experiments.

## Supplementary Material

Additional file 1 Additional file 1. Supplemental information and graphs Click here for file
